# COVID-19’s impact on real estate markets: review and outlook

**DOI:** 10.1007/s11408-021-00384-6

**Published:** 2021-03-25

**Authors:** Nadia Balemi, Roland Füss, Alois Weigand

**Affiliations:** 1 Swiss Institute of Banking and Finance (s/bf), University of St.Gallen (HSG), Unterer Graben 21, 9000 St. Gallen, Switzerland; 2 Centre for European Economic Research (ZEW), Mannheim, Germany; 3 Center for Real Estate and Environmental Economics, NTNU Business School, Trondheim, Norway

**Keywords:** COVID-19, Commercial real estate, Residential housing, Mortgages, G15, G19, R30

## Abstract

As symbolized by vacant office buildings, empty shopping malls and abandoned flats in metropolitan areas, the new coronavirus disease 2019 has severely impacted real estate markets. This paper provides a comprehensive literature review of the latest academic insights into how this pandemic has affected the housing, commercial real estate and the mortgage market. Moreover, these findings are linked to comprehensive statistics of each real estate sector’s performance during the crisis. Finally, the paper includes an outlook and discusses possible future developments in each real estate segment.

## Introduction

The failure to contain a new respiratory disease, named coronavirus disease 2019 or COVID-19, results in a global pandemic in March 2020. As an ultimate resort, governments worldwide respond by imposing lockdown as well as stay-at-home orders to prevent a collapse of healthcare systems. In the following months, vacant office buildings, home offices, empty shopping malls and promenades, closed restaurants, silent bars as well as clubs become symbols of social distancing and the limitation of interactions among people. Although these restrictions are effective in controlling the spread of the disease, shutdowns go in hand with a global economic crisis despite a wide range of economic support measures. Alike many industries, real estate markets, including residential and commercial real estate, as well as mortgage markets are confronted with unprecedented challenges. The number of commercial and residential property sales drops, people abandon their apartments in metropolitan areas, and households occur payment difficulties in redeeming their mortgages among others. Hence, economists and industry experts have an interest in better understanding the pandemic’s effect on real estate as well as mortgage markets and to use these insights to project the economic impact of further waves of COVID-19 in the short and long run. Especially at the end of 2020, these conclusions are valuable as many different countries, mainly in Europe, start to reimpose restrictions in order to flatten the second wave of infections.

**Fig. 1:**
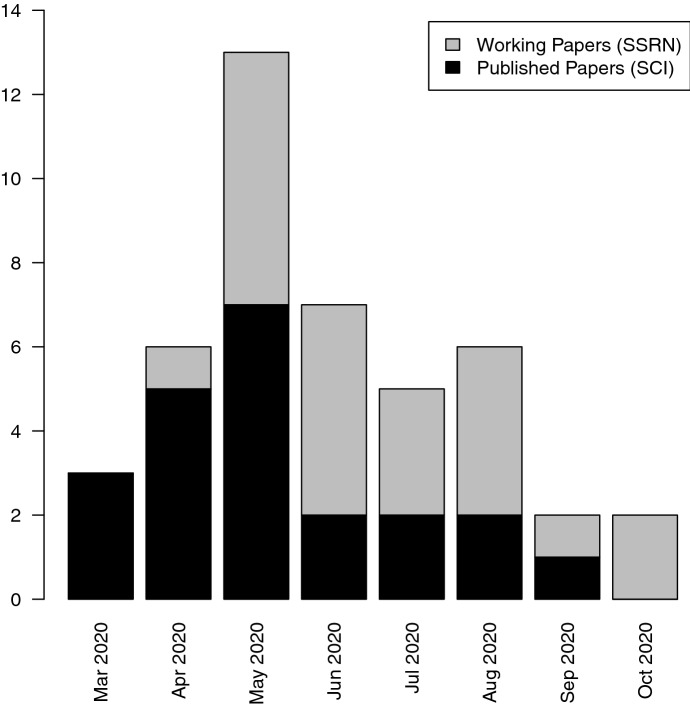
This figure depicts the numeric evolution of scientific working papers (on SSRN) and articles (in SCI ranked journals) of COVID-19’s impact on real estate markets as well as mortgage markets. Observations start in March 2020 and end in mid-October 2020

Assessing the impact of COVID-19 on real estate markets is challenging. Pandemics arrive exogenously and due to their rarity, data availability is limited, particularly due to the low frequency of real estate time series. Furthermore, it is difficult to isolate the effect on the market as prices can be significantly affected from larger macroeconomic conditions, which are temporally and geographically constrained. Nevertheless, more than 50 scientific papers appeared from March 2020 to mid-October 2020 and address this specific question. While more than half of these studies are already published in field-specific or interdisciplinary peer-reviewed journals, the other half is at a working paper stage. Figure 1 gives an overview on the number of studies over this short time period.

This paper contributes to this recent strand of literature by providing a qualitative meta-analysis. It does so by condensing the most important papers’ findings on this topic and by relating these results to relevant market indicators. Moreover, this study provides an outlook for these heterogeneous markets and discusses possible future developments and long-lasting effects of the pandemic on real estate markets. This literature review does not assess the quality of the empirical methods used in the literature.

To successfully address this literature review’s goal, the remainder of the paper is structured as follows. The second section presents an overview of the most important developments in financial markets as well as changes in macroeconomic indicators during the crisis and illustrates how these shocks are linked to the real estate and mortgages markets. The third, fourth and fifth sections start summarizing recent empirical findings on how COVID-19 affects commercial and residential real estate as well as mortgages, respectively. If available, insights from previous pandemics or epidemics are used to support or validate respective results. The sixth section provides a market outlook before the last section concludes.

## Transmission channels of the pandemic’s impact

**Fig. 2:**
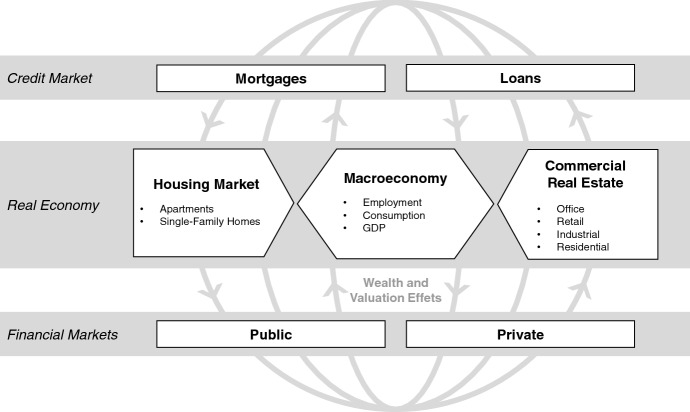
This figure illustrates the real estate and mortgage market’s connections to the macroeconomy, financial markets and other credit markets by potential wealth and valuation effects

Adapting the definition of Brodeur et al. (2020), real estate and mortgage markets can be described as a complex web of interconnected parties like households, developers, banks and investors among others. Moreover, the market is characterized by a high degree of inter-connectiveness as well as links to the overall macroeconomy and financial markets (illustrated in Fig. 2). Hence, before academic research is able to document the impact of COVID-19, it is essential to understand possible transmission channels between these parties considering the crisis’ most important developments.

At the beginning of 2020, the global economy is in a good condition. Reflected by low expected delinquency rates (Bhutta et al. 2019), households are less vulnerable compared the period leading up to the global financial crisis and businesses as well as financial corporations hold higher reserves. However, in March 2020, economic activities have been abruptly shut down and unemployment spikes as a consequence of lockdown orders. To absorb and mitigate these massive negative shifts in the supply and demand of goods at the same time, governments increase public debt and use a broad set of monetary and fiscal policy instruments^1^ such as unemployment and financial assistance programs, helicopter money, short time working benefits, mortgage forbearances, rent moratoriums for tenants and reductions in federal funds rate. Despite these measures dampening the pandemic’s effect, the global real GDP growth will still be − 5.4% (in some countries more than − 10%) in 2020 according to the OECD.

As the nexus between real estate markets and GDP growth is very persistent nowadays,^2^ declining residential and commercial property prices contributed to this fall in GDP during the pandemic alongside with economic drops in various other industries. In general, these declining real estate prices can be explained by increasing capitalization rates in the following simple valuation formula of Duca et al. (2020) where a property’s value (*V*) is given by the ratio of the properties (potential) net operation cash flows (*NCF*) and the capitalization rate:

$$\begin{aligned} V = \frac{\mathrm{NCF}}{\mathrm{Cap}\,\mathrm{Rate}\uparrow }, \quad \mathrm{with}\,\mathrm{Cap}\,\mathrm{Rate} = r_{\mathrm{f}} + \mathrm{RP }-g - \mathrm{liq}^{\mathrm{funding}}. \end{aligned}$$

The capitalization rate increases because of higher level of expected rate of returns. For example, the low interest rates ($r_{\mathrm{f}}$) during the pandemic cannot compensate a higher risk premium (RP) of owner-occupying or investing in real estate. This risk premium reflects the high uncertainty in the economy during the pandemic and a shift in risk aversion. Additionally, the expected growth in (potential) future cash flows (*g*) is lower due to COVID-19 and funding liquidity ($\mathrm{liq}^\mathrm{funding}$) dries up, increasing the capitalization rate even further. However, due to the confinement measures and the heterogeneity of real estate, these effects have different magnitudes in the case of residential and commercial real estate properties during the pandemic.

Commercial real estate sectors hit directly by the shutdown and dealing with the highest degree of uncertainty are hotel and retail properties. These buildings host businesses which have to close down entirely as they mostly provide in-person services and/or have personal points of sales. Office buildings and space for professional exchange remain vacant as well. However, businesses occupying these properties are often able to still provide their services from their employees’ home offices as they operate in industries with less need of personal contact (such as financial or professional services firms)^3^ In contrast, businesses in industrial production sites continue operating in most countries with additional safety measures and at lower capacities due to limited demand.

These negative developments are expected to create higher vacancy rates in the commercial real estate sector.^4^ In hand with a higher degree of uncertainty, these low growth expectations lead to a devaluation of commercial property portfolios of high net worth individuals, private equity funds, private as well as public real estate investment trusts or developers. As a consequence, these significant losses go in hand with higher leverage ratios and higher risk premium requirements for future investments. Due to decreasing property valuations, it becomes harder for commercial investor to get funding on the loan market. In addition, stock prices of listed real estate companies drop and dry up funding liquidity^5^ even more. Especially, firms, including real estate securities, with less cash, more debt and limited profits before 2020 show lower stock prices during the COVID-19 pandemic (Ding et al. 2020).

However, these stock price drops of real estate securities are further caused by spillovers from national as well as international financial markets and overall market uncertainty.^6^ Baker et al. (2020) argue that this uncertainty and increased volatility^7^ around COVID-19 has an even larger magnitude than the fluctuations and unpredictability associated with the global financial crisis. Moreover, Alfaro et al. (2020) precisely document how aggregate and firm-level stock returns are impacted by unanticipated changes in the predicted number of infections.

In addition, this recession on the stock market affects the wealth of households immediately. Contrastingly, monetary and fiscal policy measures are able to largely dampen COVID-19’s effect on households’ income and, hence, on residential housing markets as well as mortgage markets. However, differences across household types are present. Low-income households or minorities^8^ are more likely to be hit by unemployment or wage cuts. Workers at small firms in non-tradable sectors are more likely to be laid off (Kurmann et al. 2020), which may result in rent losses for landlords or defaulting mortgages. In the medium and long run, it will also be crucial to assess COVID-19’s impact on blue- versus white-collar workers, to understand changes in the housing and mortgage market.

Although real estate markets show quick signs of recovery during the summer months of 2020, documenting and quantifying the above effects in academic studies is essential. The second wave of infections and possible future waves until a COVID-19 vaccine is introduced will go in hand with similar restrictions, which reinforce the described negative effects further and, overall, make a financial crisis more likely to occur.

## Commercial real estate market

Assessing COVID-19’s impact on commercial real estate is difficult. In contrast to residential housing markets, data availability is limited due to a low number of transactions and the dominance of private data providers. Due to lockdown restrictions, these low transaction volumes decrease further and an analytical problem arises when assessing direct commercial real estate during the current crisis.

Therefore, van Dijk et al. (2020) analyze the gap between supply and demand as a measure for liquidity in the commercial real estate asset market for eight major cities in the USA. Their results show substantial drops in liquidity. These drops on apartment, industrial, office and retail markets are − 14%, − 14%, − 18% and − 20%, respectively, which exceed declines in liquidity during the first four months of the global financial crisis. The reason behind the large magnitude of these effects is a supply shift. According to the authors, sellers raise their reservation prices in a situation where buyers are lowering theirs resulting in a market outcome with lower volumes and no significant price changes during COVID-19.

**Fig. 3:**
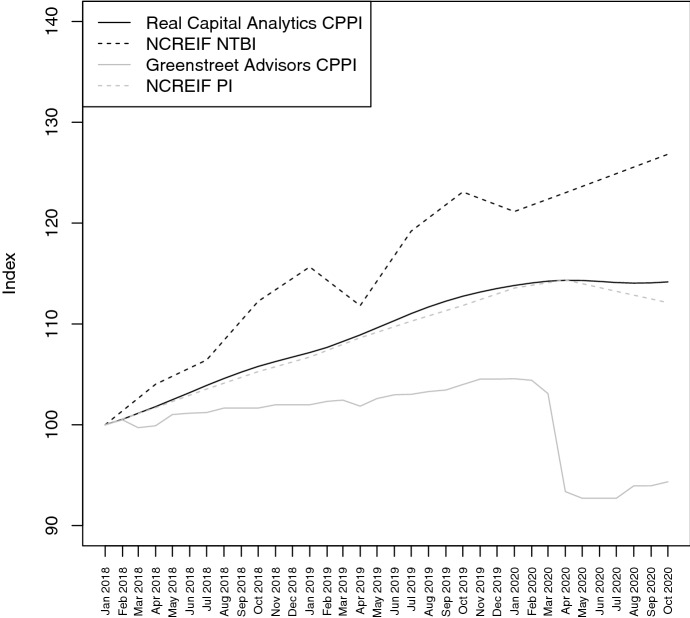
This figure compares the performance of transaction-based (Real Capital Analytics Commercial Property Price Index and NCREIF National Transaction-Based Index) to appraisal-based (Greenstreet Advisors Commercial Property Price Index and NCREIF Property Index) commercial real estate indices in the USA. The sample period spans from January 2018 to October 2020. All indices are normalized to January 2018

Figure 3 illustrates a consequence of this market outcome documented by van Dijk et al. (2020). The graph compares the developments of transaction-based indices and appraisal-based indices. During the first wave of COVID-19, transaction-based indices are either stagnating or increasing as these indices are computed based on few transactions with prices similar to the months before the outbreak. In contrast, appraisal-based indices depict price drops (smaller or larger) as these indices are based on valuations of existing portfolios.

Ling et al. (2020) document firm-level exposure of commercial real estate assets owned by listed U.S. REITs to the growth of COVID-19 cases. In this analysis, the relevant COVID-19 growth rate is computed as a weighted average of the daily growth rates of infections in counties in which the REIT owns properties. Adjusting for returns on the S&P 500 Index and the FTSE-NAREIT All Equity REITs Index, the authors find a decrease of − 0.24% and − 0.93% point in abnormal returns after a single point standard deviation increase in COVID-19 growth in a one- and three-day window, respectively. In the study of Ling et al. (2020), REITs with portfolios focused on data center, cell towers, self-storages and warehouses produce positive abnormal returns in the early stages of the outbreak. Contrastingly, hospitality and retail REITs are performing poorly.

**Fig. 4:**
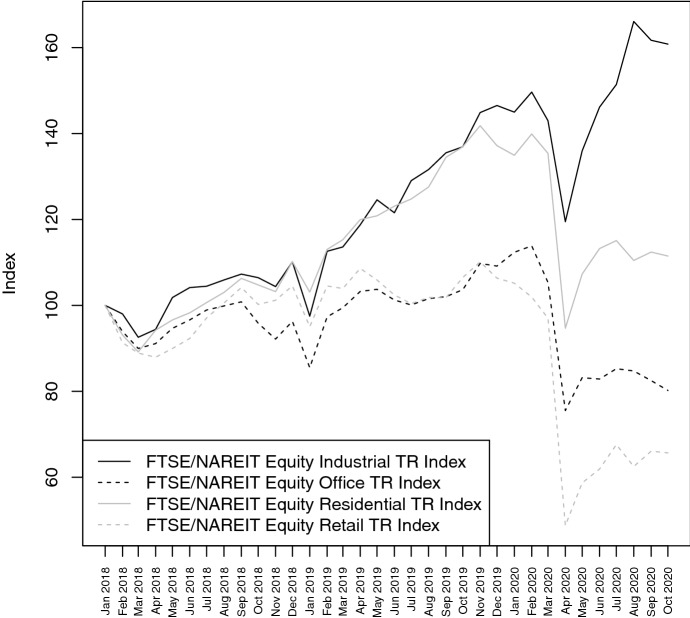
This figure compares the performance of REIT indices in the USA with different sector focus (industrial, office, residential and retail). The sample period spans from January 2018 to October 2020. All indices are normalized to January 2018

Hence, the results of Ling et al. (2020) show that commercial real estate sectors are affected differently by the COVID-19 crisis. To better illustrate this different impact, Fig. 4 depicts the price drops (and recoveries) of − 16% ($+$ 39%), − 28% ($+$ 13%), − 30% ($+$ 22%) and − 50% ($+$ 39%) for four different REIT indices in the industrial, office, residential, and retail sector, respectively. It is important to note that only the industrial REIT index raised above pre-crisis level after the first wave of infections.

Milcheva (2020) provides evidence of the pandemic’s impact on real estate securities internationally. The author employs a COVID-19 risk factor, linked to daily changes in confirmed global coronavirus infections, to capture the risk exposure in the assets’ risk-return relationship. The results show an average firm sensitivity of 0.6 with large differences across countries and sectors. Retail firms show the highest sensitivity (1.2), while healthcare stocks show the lowest (0.25). Furthermore, firms more exposed to COVID-19 suffer from a steeper decline in stock returns due to leverage constraints. As a consequence, the author argues that COVID-19 risk is predominantly propagated by financial constraints.

A study of Xie and Milcheva (2020) employs a difference-in-differences approach to identify the relation between COVID-19 cases and daily returns of real estate firms in Hong Kong during the very early stage of the pandemic. In particular, the authors develop a measure for a geographical COVID-19 footprint by identifying visited locations by COVID-19 patients as well as their homes. The author’s diff-in-diff setting bases on the fact that shutdown orders are only imposed in Hong Kong in a later stage of the pandemic. This allows investigating the impact of risks associated with the virus alone and to disentangle them from risks associated with actual building shutdowns. The authors’ most important finding argues in favor of a negative correlation. Real estate companies with a property within two miles from a COVID-19 case result in a 0.02% lower return one day after the case disclosure. This effect is stronger for buildings located closer and weaker for residential properties.

## Housing market

Several studies analyze the impact of past pandemics or epidemics on residential housing. The insights of these academic papers provide a foundation for analysis of the housing market under COVID-19.

### Housing and past pandemics

A historical-based study of Francke and Korevaar (2020) analyzes plague and cholera outbreaks in Amsterdam and in Paris during the sixteenth–seventeenth and nineteenth century, respectively. The authors document significant yearly losses for aggregate house prices of about − 6% on average until one year after an epidemic. Rents follow the same pattern. However, the decline in rents is limited to − 3% per year. Furthermore, sales prices for houses one year after an epidemic are 13% lower and the decline in house prices is 10% larger when doubling cholera mortality in a specific neighborhood. The results highlight stronger declining prices in highly affected areas (especially at the beginning of an epidemic). Lastly, the authors describe these declines as transitory shocks, as both cities could revert to their initial price path after the epidemic.

Ambrus et al. (2020) investigate the historical footprint of epidemics on cities by looking at the impact of one cholera outbreak in one residential area of London during the nineteenth century. During this epidemic, 660 residents in a 0.5-mile radius from St. James Parish died within a month. The authors show that ten years after the pandemic, rental values are 15% lower within the catchment area of the water pump that transmitted the disease. Surprisingly, compared to similar neighborhoods, the difference in house prices persists over the following 160 years as the tenant composition within the neighborhood changed. Hence, these results argue for a permanent effect on rental markets due to the exogenous character of risks of epidemics.

Wong (2008) investigates how the price of individual residential properties is affected by the SARS outbreak in Hong Kong in 2003. The epidemic results in decreasing demand for housing, rather than a decline in supply. The author confirms an average diminution of − 1% to − 3% in prices when an estate is directly affected by SARS. (Infection rates are publicly for each property and mentioned in the media.) As a consequence of the outbreak, the average total reduction in value across all estates is − 1.6%. A comparably low figure, as behavioral economic theories argue the decline in house prices should be stronger due to overreaction in cases of rare events (Viscusi 1989). Wong (2008) explains this absence of price overreaction in an analysis of transaction volumes. Despite the significant diminution in turnover rates, this analysis suggests that house prices do not overreact because housing market features in Hong Kong are characterized by high transaction costs, liquidity constraints and loss aversion.

Another study of Davis (2004) is based on the argument that houses located in regions with high risks must have lower prices than equivalent houses in locations with lower risk. In this perspective, the author analyzes the housing market for an isolated county in Nevada suffering from a severe and unexplained increase in pediatric leukemia. His results show how housing prices decline significantly as a response of increased health risk following the diagnoses of the leukemia cases. The decrease in property values reaches a maximum of approximately − 15% compared to the control group in a neighboring county. Furthermore, D’Lima et al. (2020) investigate and confirm the same argument as Davis (2004) by analyzing a dataset on opioid prescriptions as well as sales in Ohio during the “opioid crisis”. This health crisis refers to overuse and misuse of prescription opioids from the late 1990s. Their findings demonstrate dropping house prices around dispense locations (pharmacies and practitioners) and a negative correlation with the quantity of opioids dispensed.

### Housing and COVID-19

A first decisive documentation of the impact of the COVID-19 pandemic on the housing market is the work by D’Lima et al. (2020). Based on a difference-in-differences framework, the authors investigate the effects of shutdown and reopening orders by looking at data of one million housing transactions between the 1 January 2020 and the 20 June 2020 in the USA, considering both states which impose and states which do not impose statewide shutdown measures. The results do not argue in favor of any aggregate price effect, while demonstrating evidence for a significant decrease in transaction volume.

**Fig. 5:**
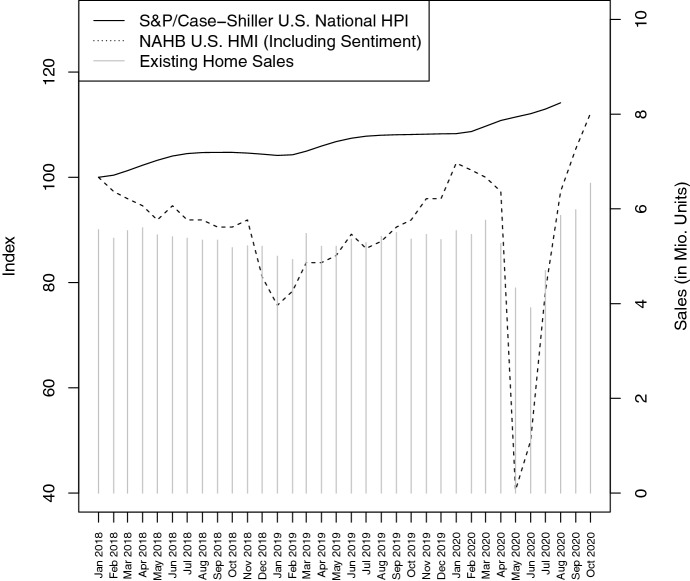
This figure compares two different house price indices in the USA (left vertical axis) and relates their performance to monthly sales of existing homes from the National Association of Realtors (right vertical axis). The NAHB U.S. Housing Market Index includes a market sentiment measure, while the S&P/Case-Shiller U.S. National House Price Index is purely transaction based. The sample period spans from January 2018 to October 2020. House price indices are normalized to January 2018

The finding of D’Lima et al. (2020) is confirmed by Fig. 5. The graph shows a continuous price increase of the Case/Shiller U.S. National House Price Index from January 2018 to October 2020 with almost no reaction to the COVID-19 crisis. In contrast, the high uncertainty in the market leads to a plunge on the supply side, where existing home sales decreases by more than − 30% between February and June 2020. Surprisingly, the market recovers quickly and reaches an all-time high in September 2020. In a similar way, the U.S. Housing Market Index of the National Association of Home Builders^9^ drops. Besides recent transaction data, this index includes market sentiment for single-family homes and shows a pessimistic decline of − 60% followed by rebounding optimism driving up the index by $+$ 177% to its all-time high.

On the one hand, D’Lima et al. (2020) explain the decrease in transactions by a shift in supply, as the number of daily listings decreased due to sellers’ risk aversion in light of considerable uncertainty dominating the market. Large properties are most illiquid under these conditions. Furthermore, Yoruk (2020) documents a decreasing number of new home listings as well as pending home sales in fifty major US cities in the early stage of the pandemic. On the other hand, many frictions in the housing market are due to demand side shocks as buyers are unable to conduct optimal search and properly process their biddings, while COVID-19 restriction orders are in place (D’Lima et al. 2020). Tanrıvermiş (2020), Bhoj (2020) as well as Jovanovic et al. (2020) document similar shifts in demand or supply of international real estate markets like Turkey, India, China, Great Britain, Serbia and Italy.

Liu and Su (2020) analyze the correlation between demand on the housing market and population density in the USA after the outbreak of COVID-19. The authors’ results illustrate how a decline in demand is stronger in more densely populated neighborhoods and central cities. Hence, the study documents a downward shift of people’s demand for density. The explanation of this effect is based on a diminishing need of living close to jobs that are telework-compatible and a decreasing utility of easy access to consumption amenities. These weaker incentives for bearing high housing costs go in hand with growing health concerns about living in high-density locations according to the authors. Furthermore, the study finds evidence for a persistent decline in demand for housing in dense areas, while the aggregate housing market seems to recover from the shock induced by COVID-19. This outcome suggests that the aversion to crowded places may preserve in the future.

A fundamental analysis of the nexus between housing stability (defined as housing tenure and security) and the success of public health strategies during COVID-19 is provided by Layser et al. (2020). The authors argue that social distancing, as the main public health tool for mitigating the COVID-19 outbreak in the USA, is a threat for housing stability as restrictions caused significant numbers of business closures as well as a rise in unemployment. Consequently, this effect will create a downward spiral: increased housing instability will undermine the public health response as social distancing becomes more difficult due to worse economic conditions. Considering increasing eviction rates, which are also likely to grow after the market rebound, this reaction is destinated to disproportionally impact racial minorities or other disadvantaged socioeconomic groups.

On a regional level, decreasing housing prices are documented by Del Giudice et al. (2020) in the Campania Region in Italy. Firstly, the authors undermine that COVID-19 affects housing markets through several channels including the closure of entire neighborhoods or cities, concerns about long-run contagion/distrust of the effectiveness of sanitation effort, general economic decline as well as specific housing market factors. Secondly, home sale declines are likely to stem from the demand side due to income and psychological (stigma) effects. Thirdly, in a short-run scenario, a housing price decrease of − 4.16% is quantified, while in the mid-run the price drop is calculated as − 6.49% until the beginning of 2021. A similar price path is projected by Allen-Coghlan and McQuinn (2020) for the Irish housing market.

Additionally, a study by Zhao (2020), takes a detailed look at the recovery of the housing market after April 2020. The author documents increasing property prices and higher housing demand due to the Federal Reserve’s unprecedented monetary easing as a part of COVID-19 fighting policies. This is confirmed by the patterns shown in Fig. 5. In contrast to Liu and Su (2020), the study of Zhao (2020) confirms these developments in house prices as well as demand and supply patterns across urban, suburban and rural areas.

## Mortgage market

**Fig. 6:**
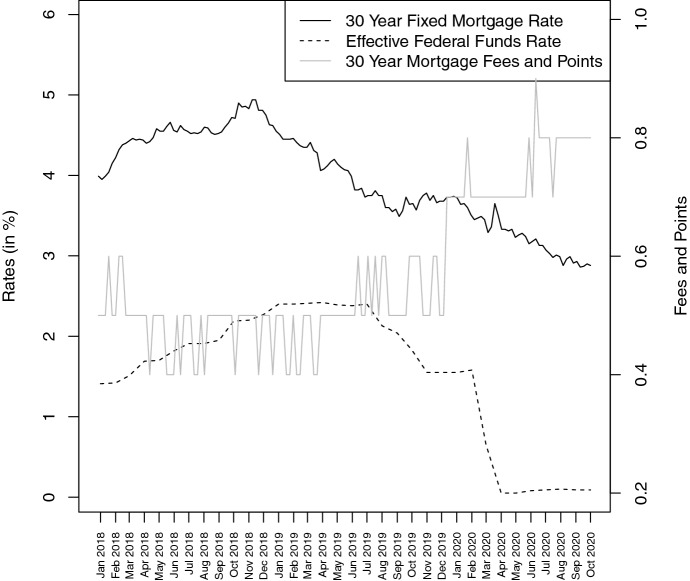
This figure compares the development of FreddieMac’s 30-year fixed mortgages rate and 30-year mortgage fees and points as well as the Federal Reserve’s effective federal funds rate. Rates refer to the left vertical axis, while fees and points are linked to the right vertical axis. The sample period spans from January 2018 to October 2020

A limited number of studies examine the situation on mortgage markets despite monetary easing programs of national banks have accelerated tremendously. This development is depicted in Fig. 6 for the USA, where the Federal Reserve decreased the effective federal funds rate to its all-time low value of 0.05% at the beginning of the COVID-19 pandemic. At the same time, the Federal Reserves pushes down the 30-year fixed mortgage rate to its record low by purchasing MBS worth USD 200 billion in total. However, as shown in Fig. 6 as well, the decreasing mortgage rates are compensated by mortgage lenders through charging higher fees and points.

Capponi et al. (2020) assess the effectiveness of regulatory responses on COVID-19 in the USA. Analyzed measures include the introduction of foreclosure moratorium, possible homeowner forbearance of mortgage payments and the Federal Reserve’s MBS purchase program. The authors document the existence of a self-reinforcing feedback loop between mortgage foreclosures and growth in house prices. Hence, an increasing number of foreclosures put a downward pressure on house prices and in turn decreasing house prices lead to more foreclosures. By modeling this feedback loop, the authors confirm that regulatory responses are successful in stopping negative spillovers from foreclosures to house prices. Nevertheless, the authors conclude that the MBS purchase program needs to target low-coupon MBS more effectively to incentives refinancing as well as home buying.

In contrast to Capponi et al. (2020), Amromin et al. (2020) highlight that in case of widespread economic disruption, like the COVID-19 pandemic, large-scale regulatory measures may place considerable pressure on the housing finance system. As an example, the authors mention the extensive use of mortgage forbearance threatening the liquidity of mortgage servicers that must replace borrower payments to investors during the early stages of the COVID-19 outbreak.

Agrawal et al. (2020) focus on the performance of commercial mortgages. By observing county-level variation in mortgage performance by property type, the authors document that the delinquency rate of commercial mortgages increases on average by approximately $+$ 3.4% for every new coronavirus case per 100 capita. In addition, the study shows no strong evidence of a decrease in delinquencies in counties with more jobs retained by the federal government’s payment protection programs relative to the local labor force. This result suggests that loans to small business do not ease the financial distress of commercial borrowers. Agrawal et al. (2020) also document within industry spillover effects at the loan level.

Another study on commercial mortgages by Griffin and Priest (2020) shows that loan valuations crucially depend on accurate loan income. However, underwritten income is commonly overstated when compared to actual property income, which in turn is highly predictive of loan distress. During the COVID-19 pandemic, the authors document appraisal aggressiveness and abnormally low capitalization rates that are related to income overstatement. Hence, the current crisis reveals “speculative” underwriting practices.

## Outlook

Predicting the medium- and long-term impact of COVID-19 on economic growth in general and on real estate markets in particular is difficult. Many factors contributing to a recovery or a potential deterioration are uncertain and dependent on the severity of the second or future waves of infections, the vaccine development as well as the governments’ will and available budget to finance additional fiscal and monetary stimulus packages. However, one aspect is certain, the longer the pandemic and shutdown orders last, the more severe will liquidity and capital risk in real estate markets become as disruptions in macroeconomic supply and demand increase further (Carlsson-Szlezak et al. 2020). Hence, the future of real estate markets is not straight forward and characterizing the shape of the recovery like Carlsson-Szlezak et al. (2020) might not be adequate or effective.^10^ Therefore, in the following we discuss potential developments or trends for the different real estate sectors and mortgage markets. These trends might conflict each other and it is unclear which trends will prevail.

As mentioned, lockdown orders affect commercial real estate sectors differently. During the COVID-19 pandemic, firms protect their employees by the implementation of home offices if possible. This shift to remote work leads to a widespread assumption that firms will continue to implement and foster home offices to an increasing degree in the future. A potential development that goes in hand with past years’ megatrend of increasing employees’ working flexibility. On the one hand, a higher degree of home offices may result in less demand for office floor space in the future. On the other hand, distance rules will necessitate even more space in terms of square meters per person and enclosed space. Additionally, the longer the commuting distance of employees the more attractive remote working gets. As a consequence, metropolitan areas and cities with bad infrastructure might be more affected by the home office trend. However, as it becomes more costly for companies to retain their employees’ productivity as well as to maintain corporate culture, co-working spaces or shared office spaces may replace fixed office floor plans in the future. These new office concepts will in turn lead to the development of mixed use of urban locations. Furthermore, the changing requirements for office space together with an overaged stock of floor space and new potential green building requirements (Pike 2020) might affect future rental prices and expected returns in the office sector.

While the stationary or non-food retail industry has been directly hit by shutdown orders, online sales increased significantly during the COVID-19 pandemic. This change in purchasing behavior is not new and reflects an acceleration of an already existing trend. Similar to the office market, it is difficult to judge how large the pandemic’s effect on retail properties will be and how the spatial distribution of retail locations across rural, sub-urban and urban areas will look like. However, the future success of retail in cities strongly depends on an even balance of traditional retail properties and increased logistic space demanded by online retailers. In particular, the presence of storage space and show rooms of online shops will be important for a timely and climate-friendly distribution of goods to consumers. This goes in hand with a change in supply chains of local manufacturing companies as the trend to buy regional products online and to support local online shops increases. Hence, strong market adjustments in the retail sector may be expected in the future. In contrast, the future of hospitality sector is still characterized by large uncertainty as it is unclear whether travel patterns (leisure and business travel) normalizes again. As a consequence, institutional investors may avoid investments in the hospitality as well as the traditional stationary retail sector in the near future and will shift their capital into logistics and the residential sector.

COVID-19’s direct impact on the housing market is relatively small. Even though households reduce their overall spending and increase savings due to a higher economic uncertainty (due to precautionary motives and liquidity preferences), low interest rates and social distancing increases the desire for privately owned homes. Moreover, the change in the working organization toward home office as well social distancing increases the demand for more living space and privacy. As an example, there may be a higher demand for single-family homes in peripheral and rural areas or holiday apartments in tourist destination, while the demand for apartments in large cities may be reduced. Contrastingly, if people keep avoiding traveling and the use of public transportation, a trend to live closer to the place of work or to commute with private means of transportation may be another possible result. Hence, it is questionable if the out-migration from metropolitan areas persists in the future.

Nevertheless, COVID-19 will impact the housing market and the mortgage market by increasing the wealth inequality between households further. In contrast to white-collar employees, blue-collar workers and low-income households will still be more affected by unemployment and pandemic uncertainty in the future. Without government support for these individuals, an increasing share of these household will not be able to pay their rents. In the market for owner-occupied housing, the picture will be similar. Mortgages of low-income households will be more likely to default, while high-income households take up more mortgages for renovations, room extensions and larger homes to accommodate a home office for example. Simultaneously, higher property prices in the past decade lowered the collateral requirements for households. However, the longer the pandemic lasts and the more prolonged the shrinkage in consumption of high-income households will be, the higher these negative effects and their impact on the mortgage market will be. In particular, the end of the credit for investors and rent moratoriums for renters may lead to increased insolvencies of landlords, as too high debt coverage ratios and loan-to-value ratios cannot be sustained. More vacant space and income losses will be the result at real estate asset markets.

Finally, the pandemic speeds up the digitalization in all real estate markets as a catalyst for the entire economy in general and the banking and real estate sector in particular. As an example, outdated information technology in the banking sector required longer loan processing times and missing transactions hinder the property valuation during the first wave of infections. These difficulties highlight the need for digitalization and the use of artificial intelligence, machine learning, data analytics and the cooperation with Fintech firms and technology providers to facilitate existing processes. The increased use of digital channels across all real estate sectors will increase market transparency as well as market efficiency.

## Conclusion

This paper provides an in-depth overview of the scientific papers related to the impact of the COVID-19 pandemic on real estate markets. These markets include the commercial real estate, residential property as well as the mortgage market. Cited papers mainly include studies from the USA but also expand to international markets like Hong Kong, Italy, or Ireland among others. Nevertheless, the reviewed research shows that due to the heterogeneity of real estate and varying transmission channels from initial macroeconomic shocks, all real estate markets are affected in different ways by the outbreak of the virus. Understanding these differences and their future consequences is crucial for governments, national banks, or investors in private as well as public markets. In particular, the summarized findings are valuable for various countries who are currently fighting the second wave of infections and those who have to prepare for potential future waves. In addition, future research should consider existing studies on COVID-19 and should dig deeper on possible links between real estate markets, the macroeconomy and financial markets.

## Footnotes

1 Contrastingly, rescue programs during the global financial crisis target on stabilizing the banking sector and reducing the systemic risk in the financial system. Measures applied during the COVID-19 pandemic aim at buffering the initial shock in the real economy and the losses of the respective market participants.

2 For instance, (Rogoff and Yang (2020)) find that a 20% decrease in real estate activity leads up to 10% fall in GDP for today’s Chinese economy, even without any divulgation through the banking system.

3 Dingel et al. (2020) classify the feasibility of working at home across industries and cities. The authors illustrate that 37% of jobs in the USA can be reallocated remotely and these jobs correspond to 46% of the national wages. Hence, these jobs typically pay more than jobs that cannot be done at home. This is in line with Bartik et al. (2020), who find evidence that remote work in industries with better paid and better educated employees is more common compared to other industries.

4 For instance, local retail businesses shifting to online sales generate significantly less demand for retail floor space. Furthermore, service companies increasingly use telecommunication and support working from home offices. In both cases, firms will cut rental space substantially and will negotiate new leases for less space.

5 This development of real estate securities as non-pandemic-resilient stocks is in line with Pagano et al. (2020). These authors find that pandemic-resilient stocks of firms, which are less affected by social distancing, substantially outperform their peers, even after controlling for the other standard risk factors.

6 In accordance with Shiller (1981) and Campbell and Shiller (1988), Gormsen and Koijen (2020) point out that most of the variation in stock values can be traced back to changes in expected returns rather than revisions in expected future growth rates. The changes in investors’ discount rates may reflect shifts in risk aversion, sentiment, or uncertainty about long-term growth.

7 Baker et al. (2020) develop an equity market volatility (EVM) tracker which is a newspaper-based indicator that moves with the VIX and the realized volatility on the S&P 500. The authors note that by February 2020, COVID-19 infections start to dominate newspaper coverage of stock market volatility and newspaper discussions on economic policy uncertainty. In March 2020, COVID-19 developments receive attention in more than 90% of all newspaper discussions of market volatility and policy uncertainty.

8 By implementing a difference-in-differences setting, Verhaeghe and Ghekiere (2020) show how ethnic discrimination in a Belgian city’s housing market increases during the COVID-19 pandemic. The authors identify lower income levels as one possible reason for this discrimination.

9 The NAHB HMI is based on transaction prices as well as on a monthly survey of NAHB members in order to capture the sentiment in the single-family housing market. The respondents of the survey rate the market conditions for new home sales “at the present time and in the next six months as well as the traffic of prospective buyers of new homes”.

10 Carlsson-Szlezak et al. (2020) list three exemplary shapes of economic shocks: the V-shape with a significant drop in GDP and fast recovery back to the pre-crisis growth path, the U-shape with a strong drop in growth never reaching the pre-pandemic path and the L-shape with not only recovering but also further declining growth rates. Undoubtedly, the first wave of the pandemic followed a deep but short pattern. With more waves of COVID-19 infections, it is likely that the growth path of economic development shows a deep L- or U-shape.
